# Topical Tranexamic Acid and Chest Masculinization Surgeries—Impact on Postoperative Hematoma Incidence

**DOI:** 10.1016/j.jpra.2025.01.002

**Published:** 2025-01-10

**Authors:** Krisztina Sipos, Katriina Joensuu, Susanna Kauhanen, Kaisu Ojala

**Affiliations:** a Department of Musculoskeletal and Plastic Surgery, University of Helsinki, and Helsinki University Hospital, Finland; b Department of Plastic Surgery, Tampere University Hospital, Finland

**Keywords:** Transgender, Masculinization, Breast surgery, Tranexamic acid, Hematoma, Postoperative complications

## Abstract

**Introduction:**

Postoperative hematoma requiring intervention occurs more frequently in chest masculinization surgeries than in other types of breast surgeries, with incidences ranging from 0.7% to 13.2% per patient. Although there is increasing evidence that topically applied tranexamic acid (TXA) effectively reduces postoperative bleeding in breast surgeries, its impact on masculinization surgeries is understudied.

**Aims:**

Examining the significance of topical TXA in reducing postoperative hematoma in chest masculinization surgeries.

**Methods:**

This retrospective cohort comprises female-to-male transgender and non-binary patients who underwent chest masculinization at Helsinki or Tampere University hospitals between 2018 and 2024. Topical TXA (20 mg/ml, 25 ml per breast) was incorporated into routine use in October 2022, replacing the previous practices; Helsinki mainly operated without TXA, whereas Tampere routinely used intravenous (i.v.) TXA.

**Results:**

A total of 198 patients undergoing chest masculinization surgery were included. Among them, 9 (4.5%) major hematomas occurred. The need for reoperation due to postoperative hematoma was lower in the topical TXA (3.2%, 2 out of 63 patients) and i.v. TXA (3.4%, 2 out of 58 patients) groups compared to the non-TXA group (6.5%, 5 out of 77 patients). Subpectoral incisions (71.2%, 141 patients) resulted in a 5.0% hematoma rate, whereas periareolar incisions (28.8%, 57 cases) had a 3.5% hematoma rate.

**Conclusions:**

Our study suggests that topical and i.v. TXA effectively reduce postoperative bleeding in chest masculinization surgeries, with similar outcomes between the 2 methods. Albeit our results lack statistical significance and they support the potential benefit of prophylactic TXA use in hematoma reduction.

## Introduction

Increasing societal acceptance of gender dysphoria has led to a growing number of gender reassignment surgery requests among transgender and non-binary individuals.^1^^,^^2^ Chest wall masculinization is typically one of the initial and crucial steps in altering the physical appearance of female-to-male (FtM) transgender patients.^3^ This surgical procedure involves the removal of most breast tissue while preserving the nipple and aiming for a masculine appearance. To achieve the desired outcome, various surgical techniques can be used, depending on the initial breast volume, excess skin, ptosis, skin elasticity, and the size and position of the nipple-areola complex (NAC).^4^, ^5^, ^6^, ^7^, ^8^ Postoperative hematoma is the most common immediate complication in chest masculinization surgeries.^9^ The incidence of hematomas requiring evacuation is known to range from 0.7% to 13.2% per patient.^3^^,^^6^, ^7^, ^8^^,^^10^, ^11^, ^12^, ^13^, ^14^, ^15^, ^16^, ^17^, ^18^, ^19^, ^20^, ^21^

Tranexamic acid (TXA) is a synthetic lysine analog that inhibits fibrin clot dissolution by blocking plasmin activation, thereby preventing excessive bleeding.^22^^,^^23^ Moreover, the topical administration of TXA for bleeding prophylaxis has recently gained attention with increasing evidence of its effectiveness in plastic surgery procedures.^24^, ^25^, ^26^, ^27^ Our previous retrospective study demonstrated a tenfold reduction in postoperative hematomas with topical TXA application among reduction mammaplasty patients.^28^

### Aim of the study

Our primary aim was to investigate the efficacy of intraoperatively applied topical TXA in reducing postoperative hematomas in chest masculinizations surgeries. Secondarily, we aimed to examine the incidence of hematomas in relation to demographic and medical data, as well as various chest masculinization techniques, among the transgender and non-binary populations.

## Materials & methods

### Included patients

This retrospective cohort study involved FtM transgender and non-binary (ICD-10 codes F64.0 and F64.8) patients who underwent bilateral chest masculinization surgeries at the Plastic Surgery Departments of Helsinki University Hospital and Tampere University Hospital between January 2021 and January 2024. Our dataset was supplemented with another cohort of consecutive patients undergoing similar surgeries at Helsinki University Hospital in 2018. The study size was initially calculated based on hematoma frequency from our previous study, but it was naturally limited by the specific patient group in our sparsely populated country.

### New protocol for initiating topical TXA application

Intraoperative application of topical TXA was integrated into the hospital protocol for chest masculinization surgeries in October 2022. Following the protocol implementation, surgeons gradually adopted the TXA rinsing method during procedures—a decision that ultimately depended on the preference of the individual surgeon. TXA was applied directly to the breast wound from a syringe and rinsing the wound before closure. This practice supplanted previous practices, wherein TXA was not commonly used as a prophylaxis in the Helsinki unit, while in the Tampere unit, intravenous (i.v.) TXA was routinely applied. The TXA rinse was used irrespective of whether the patient had participated in the study; rather, it is an integral part of the hospital protocol, based on previous positive outcomes.

### Hormone therapy

In Finland, typically, FtM patients are required to undergo at least 12 months of testosterone therapy prior to chest masculinization surgeries, and it is not interrupted by the surgery. Younger trans men may use gonadotropin-releasing hormone analogs as the preliminary step before starting testosterone as part of their hormone therapy to suppress estrogen production and achieve a more masculine hormonal profile.

### Surgical technique

**Figure 1:**
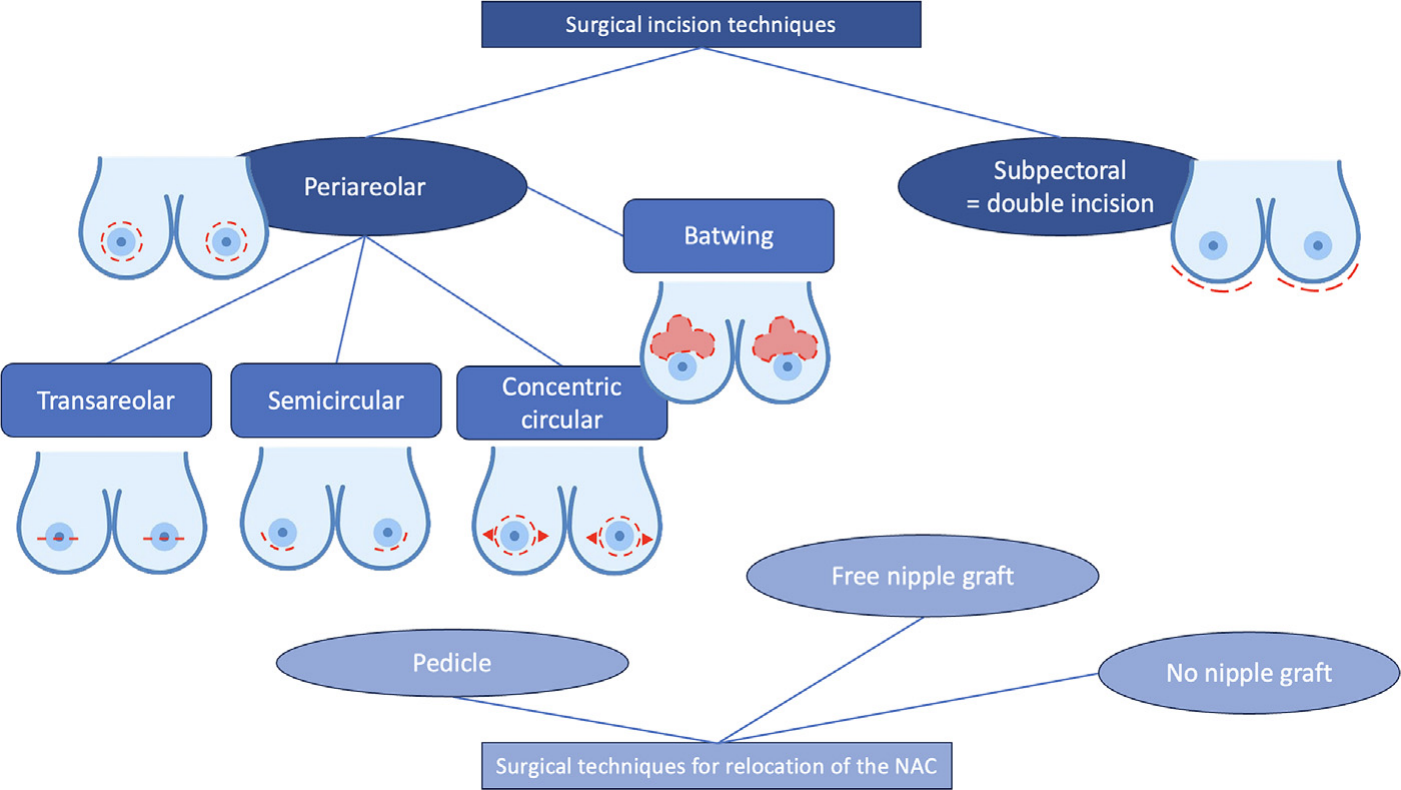
Surgical techniques.

**Figure 2:**
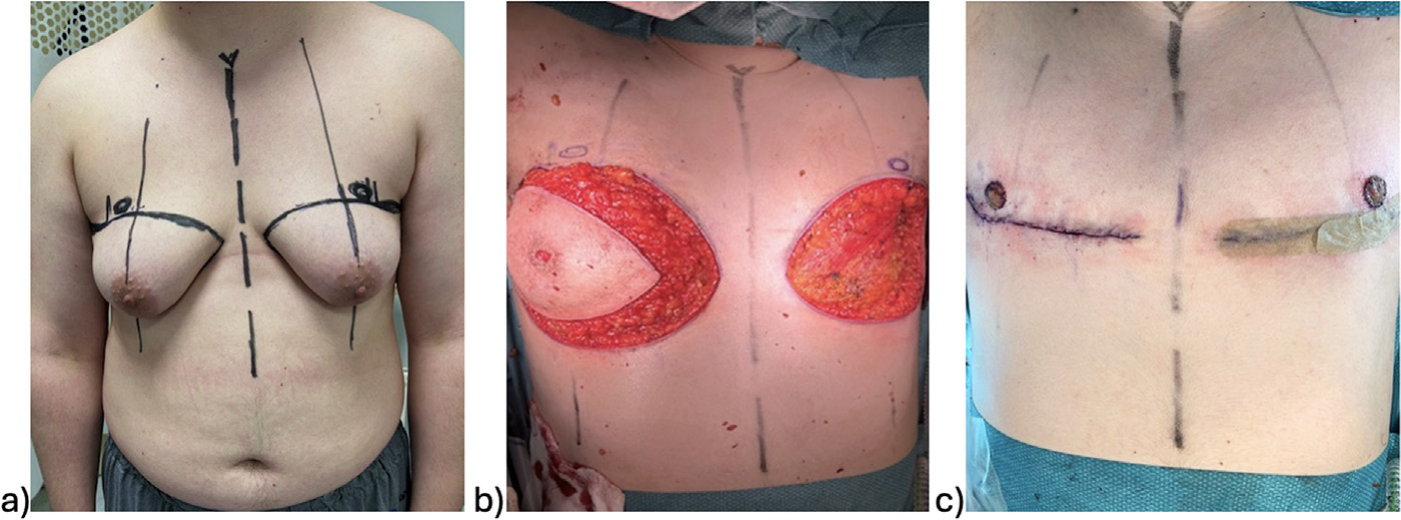
Subpectoral incision and free nipple grafts; a) preoperative, b) intraoperative, c) postoperative.

**Figure 3:**
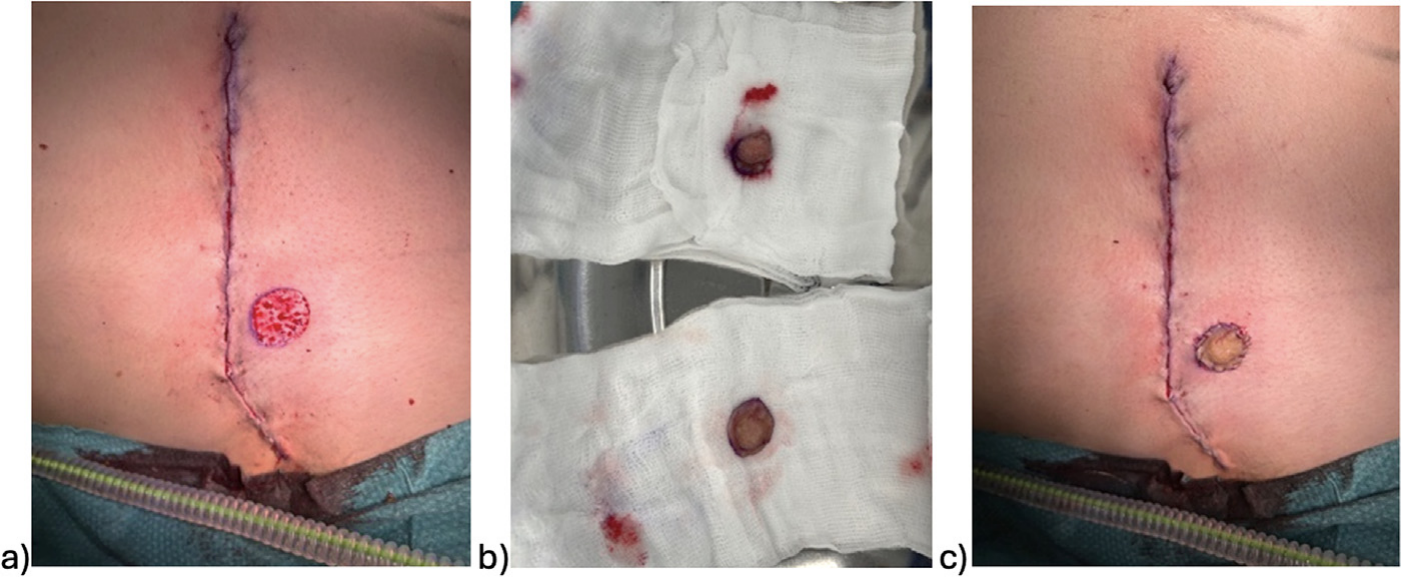
Free nipple graft.

**Figure 4:**
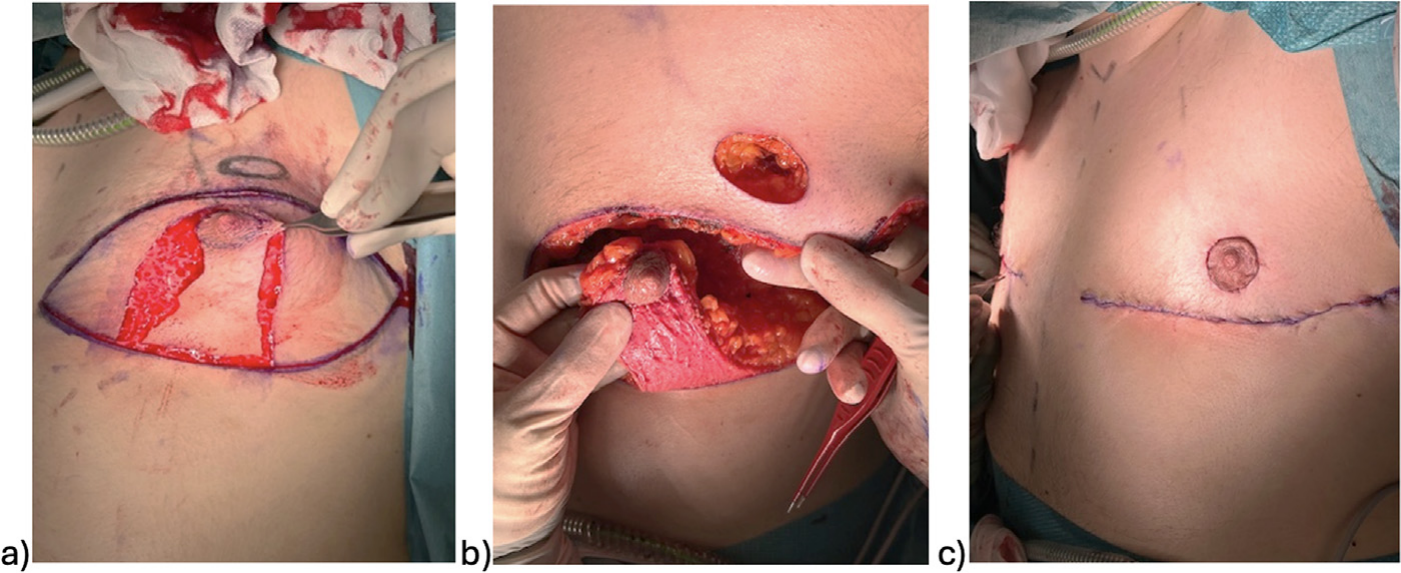
Subpectoral incision and relocation of the nipple-areola complex (NAC) with a pedicle; a) and b) intraoperative, c) postoperative.

The surgical procedures were primarily conducted by plastic surgeons. Chest masculinization involves various techniques for incision and relocation of the NAC. Incision techniques can be broadly categorized as periareolar and subpectoral (Figures 1–4). NAC relocation can be achieved by using a pedicle, a free nipple graft, or opting not to place new nipples (Figure 1, Figure 2, Figure 3, Figure 4). Several other pedicle techniques were used but they have not been reported separately. In selecting the surgical technique, individual breast characteristics, surgeon preference, and hospital protocols played a role. Patients’ preferences regarding surgical incisions were also considered during the preoperative discussions. No specific algorithm was employed for decision-making.^4^

### Intra- and postoperative protocols

All patients were placed under general anesthesia. After dissection, hemostasis was achieved while aiming to maintain systolic blood pressure at 120 mmHg or at least 100 mmHg. TXA was administered directly to the open breast wound before closure, at a dose of 25 ml per breast at a concentration of 20 mg/ml. Alternatively, if i.v. TXA was used, the dose was 1 g TXA (10 ml of a 100 mg/ml solution). At the end of the surgery, most patients received long-acting local anesthetic, ropivacaine, to alleviate postoperative pain. The use of drains over time has evolved from standard routine practice to an exception, and it ultimately depends on the surgeon's discretion. After the wounds are closed, a compression garment is routinely applied. Thromboprophylaxis after these surgeries is rare and used only if patient-related risk factors are present.

### Pre, intra, and postoperative parameters

We collected preoperative parameters including patient age, body mass index (BMI), ASA classification, hormonal therapy details, and factors affecting coagulation. Intraoperative data comprised surgical technique, resection weight, thromboprophylaxis, drain usage, and TXA administration. Postoperative parameters included hematoma evacuation, seroma, wound dehiscence, fistula, NAC necrosis, infection, scar problem, and corrective surgery. Detailed information, quantities, and distributions between groups can be found in Table 1, Table 2.

**Table 1:** Pre-, intra-, and postoperative parameters.

***Indicates too few instances to calculate a reliable p-value. * Indicates missing data on some patients.

**Table 2:** Postoperative complications.

*** Indicates too few instances to calculate a reliable p-value. * Indicates few patients (3-5) with missing data due to a lack of follow up. An additional 19 patients were missing data on corrective surgeries because the one-year follow-up with the surgeon is yet to be completed.

### Classification of postoperative hematomas

**Figure 5:**
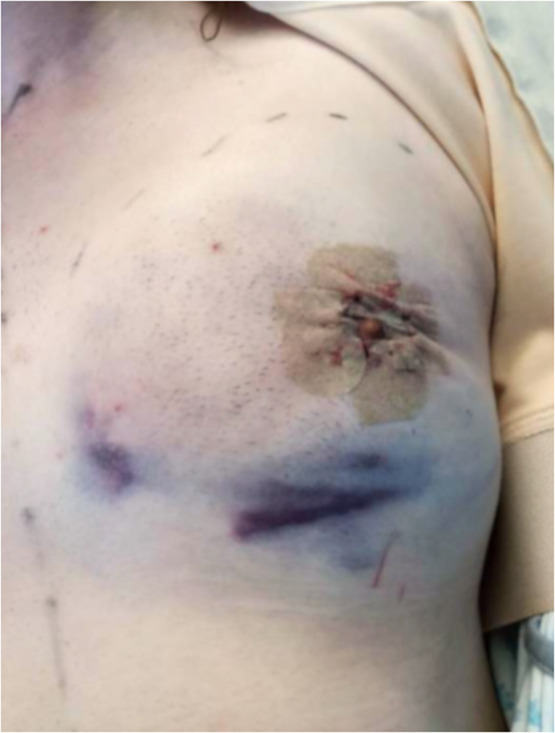
Postoperative hematoma before evacuation.

Postoperative hematomas were categorized based on the required treatment. Hematomas necessitating emergency evacuation in the operating room were considered major. Minor hematomas were conservatively managed. In this study, we focused on the incidence of hematomas requiring evacuation. (Figure 5) The comparison of hematoma incidence between groups was primarily based on major hematomas, owing to the difficulty in reliably documenting all conservatively managed minor hematomas retrospectively.

## Results

### Study groups

This study involved patients who underwent chest masculinization surgery. One hundred thirty-five (67.8%) patients were operated on in Helsinki, while the remaining 64 patients (32.2%) were operated on at Tampere University Hospital. One patient who received TXA intraoperatively in intravenous and topical forms and was omitted from the analysis. Totally, 198 patients were included and categorized into 3 main groups based on the TXA administration method. The topical TXA group comprised 63 patients (31.8%), intravenous TXA group included 58 patients (29.3%), and non-TXA group consisted of 77 patients (38.9%) (Table 1).

### Demographic data and differences between groups

All the 3 study groups were overall similar and comparable with some exceptions (Table 1). Significant differences were found in BMI and resection weight, with higher values in the i.v. group. Overall, 96.5% received testosterone therapy before surgery. Among them, 12 patients (6.3%) received <12 months of treatment. Subpectoral incisions were predominant (71.2%–141 patients) in all groups, whereas periareolar incisions were used only in 57 cases (28.8%), mainly in the non-TXA group. NAC relocation techniques were differently distributed; for example, in the i.v. TXA group the free nipple graft technique was more favored. Drains were mostly used in the non-TXA group. Approximately, half (46.5%) of the patients were discharged on the same day of surgery. In the non-TXA group this was rare, whereas in the i.v. TXA group outpatients dominated. Table 1 summarizes the demographics and patient characteristics.

### Postoperative hematomas

Nine postoperative hematomas necessitating evacuation (4.5%) occurred among a total of 198 patients. In the topical TXA group (comprising 63 patients) 2 hematomas (3.2%) were noted, and the i.v. TXA group (comprising 58 patients) also had 2 hematomas (3.4%). In contrast, the non-TXA group (77 patients) had 5 hematomas (6.5%) (Flowchart 1). The use of TXA reduced the number of postoperative hematomas requiring evacuation by half, although the differences did not reach statistical significance (p=0.46).

When considering the minor hematomas that were managed conservatively, the topical TXA group had 3 small hematomas (4.8%) compared to 4 (6.9%) in the i.v. TXA group and 6 (7.8%) in the non-TXA group (ns), paralleling the findings of major hematomas (Table 2).

### Other postoperative complications

Wound healing problems such as dehiscence and stitch fistula appeared in 45 cases (22.7%) (Table 2). There was no statistically significant difference in minor wound healing problems between TXA study groups (p=0.062). Infections requiring antibiotic treatment were uncommon and occurred in 8 patients (4.0%), with even distribution between the topical TXA and non-TXA groups (Table 2). No severe infections or revision due to infection were observed following these surgeries.

Seromas were the second most common complication that occurred in 19 cases of the 198 cases (9.6%) (Table 2). Seventeen of them needed at least one aspiration. Seromas tended to occur more frequently after topical TXA rinses (10 cases, 15.9%) compared to the non-TXA (5 cases, 6.7%) or i.v. TXA (4 cases, 6.9%) groups in this series. However, this difference was not statistically significant (p=0.12). Two out of 9 patients who had major hematoma post-operatively developed a seroma later during the healing process.

Among the 12 cases (6.1%) of NAC necrosis, only one case occurred after a hematoma. Two cases required revision due to complete NAC necrosis, whereas the remaining cases, which involved only superficial NAC necrosis were conservatively managed. Eight of these were operated using the pedicle technique, while the remaining 4 underwent surgery with a free nipple graft. NAC relocation techniques did not appear to affect NAC necrosis incidence (ns).

### Factors that may influence coagulation and hematoma formation

With the subpectoral incision, major hematomas were observed in 7 patients (5.0%). Among them, 4 did not receive TXA, 2 received i.v. TXA, and 1 received topical TXA. Regarding the periareolar approach, 2 cases had hematomas (3.5%); 1 received topical TXA, while the other did not receive TXA in any form. (Flowchart 1) There were no significant differences between incision or NAC relocation techniques with respect to the incidence of postoperative hematoma.

**Flowchart 1:**
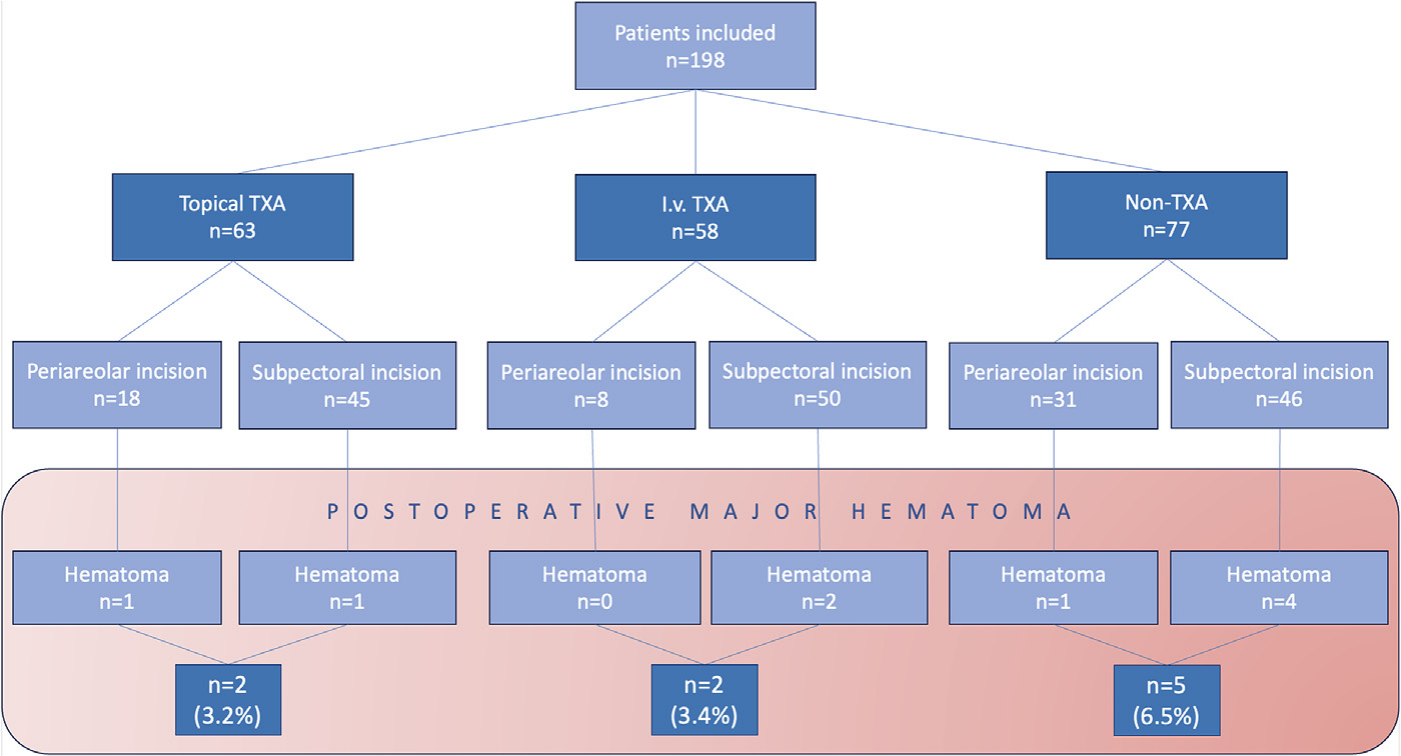
Distribution of postoperative major hematomas among the different subgroups.

Although several patients had mental health disorders diagnosed during the initial assessments, only 34 patients (17.2%) used antidepressant medication (Selective Serotonin Reuptake Inhibitors (SSRIs), Serotonin-Norepinephrine Reuptake Inhibitors (SNRIs), duloxetine, and venlafaxine) at the time of surgery. Antidepressant users had a higher percentage of postoperative hematoma (8.8%) compared to non-users (3.7%), although the difference was not statistically significant.

Only a few patients had a known tendency for bleeding or thromboembolic events, and none of them experienced postoperative hematoma. Two patients with known bleeding tendencies received i.v. TXA treatment during surgery. Three patients had thromboembolic tendencies: 2 in the non-TXA group and 1 in the topical TXA group, 2 of these patients received thromboprophylaxis after surgery. Overall, thromboprophylaxis use was rare and was administered to only 6 patients, and none of them experienced postoperative bleeding. Omega-3 use was not associated with an increased risk of postoperative hematoma in this study, but the sample size of omega-3 users was limited. None of the patients were taking anticoagulant medication.

Drains were used in 88 cases (44.4%) and 5 hematomas were reported among these patients (5.7%). We could not demonstrate any statistical correlation between drain usage and postoperative hematoma. Over time, there has been a decrease in drain usage. In 2018, all but one (96.6%) of the total 29 patients underwent surgery with drains inserted. By 2021, only 56.2% had drains, and the decreasing trend continued in 2022, with only 39.5% of patients receiving drains. After 2023, drains became remarkably rare, with only 6.8% having them (4 out of 59 patients). Most patients in the non-TXA group received drains (76.6%), as these surgeries were performed earlier in the study period. In contrast, drain usage was less common in the i.v. (29.3%) and topical TXA (19.0%) groups.

#### Corrective surgeries and scars

Fifty-five patients (27.8%) required corrective surgery later on, and 10 of them are still waiting for corrective surgery. Nineteen patients have not yet been followed-up to determine their need for corrective surgery. The indications for corrective surgeries included wound openings, NAC necrosis, excess skin or subcutaneous tissue, scar hypertrophy, breast asymmetry, or nonoptimal NAC form (Table 3). Three out of 9 patients who experienced postoperative hematomas required subsequent corrective surgery, but this did not represent a significantly higher need for corrections compared to patients without hematomas. The periareolar incision required corrective surgery more frequently than subpectoral incision. Incision type or postoperative hematoma did not have a significant impact on late-stage scarring.

**Table 3:** Need for corrective surgeries classified by different surgical techniques.

## Discussion

As the number of gender reassignment surgeries increase, there is a growing need to raise awareness and conduct research on factors that can minimize the occurrence of potential complications. The rate of postoperative hematomas requiring evacuation was comparatively high following chest masculinization surgeries. This trend is speculated to result from several factors, including various mastectomy techniques, hormone replacement therapies, and the use of other commonly prescribed medications among transgender patients; however, none of these factors have been definitively shown.

### Hematoma rate and TXA administration

The hematoma rate in our study was comparable to previously reported numbers. The aim of the study was to demonstrate the potential anti-bleeding effect of topically administered TXA as a routine prophylaxis. Postoperative bleeding decreased by half, from 6.5% to 3.2%, following the introduction of topical TXA to the routine protocol compared to not using TXA. However, we did not achieve statistical significance in this regard, possibly owing to the low number of hematomas.

We recommend topical TXA over i.v. administration because both methods appear to have similar efficacy; however, topical administration has been shown to have negligible systemic absorption.^29^ I.v. administration, contrastingly, involves higher systemic concentrations and has been associated with adverse effects such as neurological convulsions and thromboembolic events.^30^^,^^31^ Although the risk of systemic adverse effects from i.v. TXA has been reported to be relatively low, routine systemic administration may add to the risks.

### Impact of surgical technique

In general, periareolar nipple-sparing techniques have been associated with more hematomas than the subpectoral technique with free nipple grafts and decreased visibility during the hemostasis has been suggested as an explanation for this trend.^8^^,^^13^^,^^17^^,^^19^^,^^32^ Diverging from previous studies, our study showed no significant difference between surgical techniques and postoperative hematomas. Interestingly, in contrast to earlier findings, our study revealed a slightly higher incidence of hematomas in subpectoral incisions (ns), potentially due to the predominant use of this approach among our patients.

### Use of drains over time

Drain-free mastectomies have lately been widely advocated.^10^ Consequently, routine clinical practices have shifted in that direction, as observed in our study. According to the clinical assumption, if a true postoperative hematoma requiring evacuation occurs, a drain alone cannot resolve the situation.

### Effect of testosterone on bleeding

There is no clear evidence from other studies suggesting a connection between testosterone use and the development of hematoma after chest masculinization surgeries.^18^ In our series, most patients were under testosterone therapy at the time of surgery, which prevented us from making reliable statistical comparisons.

### Effect of antidepressant medication on bleeding

Generally, antidepressants, especially serotonergic ones (SSRIs and SNRIs), have been associated with an increased risk of bleeding.^33^^,^^34^ This risk is particularly evident when they are used in combination with nonsteroidal anti-inflammatory drugs (NSAIDs)^35^, which are also commonly prescribed after masculinization surgery. We observed a trend between antidepressant use and hematoma formation, which was consistent with those in previous studies (ns). Hence, if the patient is on antidepressant medication, NSAIDs should be used with caution.

### Seroma

In previous studies, seroma rates in chest masculinization surgeries ranged from 0% to 7%.^10^^,^^11^^,^^19^^,^^32^ The overall seroma prevalence was slightly higher, 9.6% in our series, with the topical TXA group showing the highest incidence (15.9%) (ns). In contrast, our previous study on reduction mammaplasties found a considerably lower seroma rate of 2.2%, with the topical TXA group having lower prevalence.^28^ Ausen K et al. in 2020 ^36^ showed no overall significant difference in seroma formation in topical TXA and placebo in breast cancer patients undergoing mastectomy. However, subgroup analyses found an increase in seroma formation in TXA patients who had also undergone axillary dissection and they postulated that topical TXA might not be beneficial for reducing lymph leakage. This discrepancy warrants further investigation and should be considered in future prospective studies.

### Strengths and limitations of the study

The retrospective nature of our study is a limitation. Patients in the non-TXA and i.v. TXA groups underwent surgery at an earlier time, while those who received topical TXA had more recent surgeries. This presents a challenge in accounting for changes in surgical practices over time. The multicenter aspect of our study is a strength and a limitation, as the utilization of TXA protocols varied between hospitals before the routine introduction of topical TXA. When considering late-onset postoperative complications, not all patients followed the same treatment and follow-up protocols, reflecting the differences between hospitals. Notably, the 2 largest transgender surgery centers in Finland successfully collaborated to conduct this multicenter study. However, a multicenter prospective randomized controlled trial is underway, providing an opportunity pause and reflect on the current trends. The findings of this study, such as the variability in practices across centers and preliminary data on key outcomes, such as reoperation rates and postoperative bleeding in this patient group at our units, have been crucial in guiding the design and focus of the prospective trial.

## Conclusion

Topical TXA may offer comparable efficacy to intravenous administration, potentially providing a safe and effective alternative to current practices. Although further research is warranted, these results support the rationale for the prophylactic use of TXA in chest masculinization surgeries and offer a promising avenue for further research in the field.

## Declaration of competing interest

None.
